# Investigating the impact of *Premolis semirufa* caterpillar bristle toxins on human chondrocyte activation and inflammation

**DOI:** 10.1371/journal.pntd.0012816

**Published:** 2025-02-10

**Authors:** Cinthya Lais de Lima, Paula C. Pohl, Isadora M. Villas-Boas, Giselle Pidde, Denise V. Tambourgi

**Affiliations:** Immunochemistry Laboratory, Instituto Butantan, São Paulo, Brazil; North South University, BANGLADESH

## Abstract

**Background:**

The caterpillar of *Premolis semirufa*, known as Pararama, is found in the Brazilian Amazon, primarily on rubber trees of the genus *Hevea*. Pararamosis is an inflammatory disease resulting from accidental contact with the caterpillar’s bristles, leading to acute and chronic symptoms. Chronic exposure can cause significant osteoarticular deformities, similar to those seen in osteoarthritis and rheumatoid arthritis, due to cartilage degradation and synovial inflammation. Currently, there are no specific treatments for Pararamosis, and research on the molecular mechanisms of the caterpillar’s venom and its role in disease pathogenesis is limited. The chronic changes in Pararamosis are thought to be linked to chondrocyte activation and the NF-κB signaling pathway, influenced by the toxic components in the bristles. Understanding these interactions is crucial for developing preventive measures and therapeutic strategies, especially for rubber tappers at risk in the Amazon region.

**Methodology/principal findings:**

This study investigated the effects of *P. semirufa* bristle extracts on human chondrocytes, focusing on the activation mechanism of the NF-κB transcription factor and the expression of osteoarthritis markers. Cell viability tests indicated that the extracts did not significantly affect chondrocyte survival. However, supernatant analysis revealed a time- and dose-dependent increase in IL-6 and IL-8 levels. Additionally, the expression of NF-κB and its inhibitor, IκB, was assessed, showing higher levels of phosphorylated IκB, which induces its proteosomal degradation, compared to the negative control, while native IκB expression was greater in the control group. Furthermore, the gene expression profile of treated chondrocytes demonstrated modulation in matrix metalloproteinases (*MMPs*), aggrecan (*ACAN*), collagen type II (*COL2A1*), interleukins (*IL6* and *IL8*), and complement system molecules.

**Conclusions/significance:**

These findings highlight the significant impact of *P. semirufa* bristle extracts on human chondrocyte activation and the inflammatory processes associated with pararamosis.

## Author summary

In our study, we investigated the caterpillar *Premolis semirufa*, known as Pararama, which poses a health risk in the Brazilian Amazon, particularly for rubber tappers. Contact with its bristles can lead to Pararamosis, an inflammatory condition that may cause long-term joint issues similar to osteoarthritis. Currently, there are no specific treatments for Pararamosis, making it essential to understand how the caterpillar’s venom affects human health. We focused on how extracts from its bristles influence human cartilage cells, which are crucial for joint function. We discovered that these extracts increase inflammatory markers in cartilage cells without significantly affecting their survival. This indicates that the bristles trigger an inflammatory response that could lead to joint damage over time. By examining these effects, we aim to provide insights into the mechanisms behind Pararamosis.

## Introduction

The caterpillar of *Premolis semirufa*, commonly known as Pararama, is predominantly found on rubber trees of the genus *Hevea* in the Brazilian Amazon region. This caterpillar exhibits a dark brown coloration interspersed with red, and its body is adorned with bristles of varying sizes - small, medium, and large. Both the larva and its cocoon are covered in these bristles, which play a significant role in its interaction with humans [1–3].

The bristles of the Pararama caterpillar act like tiny spines, adhering to the human epidermis and triggering a pruritic (itchy) reaction that can worse with scratching. Incidents of contact with humans typically occur accidentally. For instance, rubber tappers, while collecting latex, may inadvertently touch tree trunks where the caterpillars are present.

Pararamosis is an occupational inflammatory disease caused by the *P. semirufa* caterpillar, manifesting in both acute and chronic forms. The acute phase is marked by an immediate urticarial reaction, while the chronic phase can lead to significant deformities due to osteoarticular changes resembling those seen in osteoarthritis [1,2,4–9]. The bristles contain trichogen cells at their base, which secrete a toxic liquid responsible for the urticarial reaction [10,11]. The chronic manifestations of Pararamosis, characterized by thickening of the synovial membrane and joint deformities typical of chronic synovitis, are often associated with repeated injury. This suggests an underlying immunological mechanism contributing to the disease’s progression.

Studies of our group have demonstrated that extracts from the bristles of *P. semirufa* exhibit potent proteolytic activity, leading to elevated antibody titers and a robust inflammatory response in murine models, characterized by the activation of macrophages and neutrophils. Furthermore, these extracts have been shown to activate T lymphocytes and antigen-presenting cells, while also enhancing cytokine production [12]. The extracts also activate complement system pathways, generating biologically active fragments as the anaphylatoxins, indicating a role for this system in the inflammatory response following envenomation [13].

Chondrocytes play an essential role in the induction of osteoarthritis, exhibiting a pro-inflammatory profile. Normally quiescent, these cells undergo a phenotypic shift in osteoarthritis, becoming activated, characterized by cell proliferation, cluster formation, and increased production of matrix proteins, matrix-degrading enzymes, and pro-inflammatory cytokines [14–18]. Studies indicate that activation of the NF-κB transcription factor is necessary to express these proteins in chondrocytes [14,15,19,20].

Nuclear factor kappa-light-chain-enhancer of activated B cells (NF-κB) is an inducible nuclear transcription factor that plays a pivotal role in regulating immune responses, inflammation, and cell survival. It is composed of various homo- and heterodimeric complexes, including NF-κB1 (p105/p50), NF-κB2 (p100/p52), RelA (p65), RelB, and c-Rel. Among these dimers, the p65/p50 complex is the most prevalent in many cell types and serves as a crucial transcription factor in cellular processes. In non-activated cells, NF-κB dimers are sequestered in the cytoplasm through their interaction with inhibitory proteins known as IκB (inhibitor of kappa B). This interaction prevents their translocation to the nucleus. Upon cellular activation, the NF-κB complex is released from IκB through phosphorylation-induced proteosomal degradation of the inhibitor mediated by IκB kinases (IKKα, IKKβ, and IKKγ). Once released, NF-κB translocates to the nucleus, where it binds to specific DNA sequences and transactivates the expression of target genes involved in various biological processes, including inflammation and immune responses [21,22].

In the context of Pararamosis, chronic osteoarticular changes may be linked to the activation of chondrocytes and the NF-κB signaling pathway by the toxic components found in the bristles of the *P. semirufa* caterpillar. Understanding the interactions between these toxic bristles and the resultant inflammatory responses is essential for developing effective preventive measures and therapeutic strategies for individuals at risk, particularly rubber tappers in the Amazon region. Therefore, this study aimed to evaluate the role of the NF-κB transcription factor in the gene activation of osteoarthritis markers in human chondrocytes treated with extracts from Pararama bristles.

## Methods

### Preparation of the extract of *P. semirufa* bristles

Caterpillars of *P. semirufa* were collected in the state of Pará, Brazil, in the municipality of São Francisco. The license for animal capture (protocol number: 11971-2) was granted by the Chico Mendes Institute for Biodiversity Conservation (ICMBIO) of the Ministry of the Environment. Access to the biological material of Pararama was authorized by the Brazilian Institute of the Environment and Renewable Natural Resources (IBAMA) (process number: 02001.008743/2011-12, authorization 01/2009) and by the National System for the Management of Genetic Heritage and Associated Traditional Knowledge (SisGen) (registration: A05C092, 11/05/2018). The caterpillar bristles were cut and transferred to tubes containing phosphate-buffered saline (PBS) (8.1 mM disodium phosphate, 1.5 mM monopotassium phosphate, 137 mM sodium chloride, and 2.7 mM potassium chloride – pH 7.4). The samples were immediately frozen at -80 °C and transferred to the Immunochemistry Laboratory at the Butantan Institute.

To prepare the extract, a bristle sample was thawed and ground using a glass rod. The insoluble material was removed by centrifugation at 2,500 × *g* for 20 minutes at 4 °C. This centrifugation step was repeated three times. The resulting supernatant was filtered through a 0.22 μm membrane (Whatman-GE Healthcare, Chicago, IL, USA), aliquoted, and stored at −80 °C. Protein concentration was determined using a bicinchoninic acid (BCA) protein assay kit (Pierce Biotechnology, Rockford, IL, USA) following the manufacturer’s instructions. The protein profile of the extract (10 µg) was examinated by sodium dodecyl sulfate polyacrylamide gel electrophoresis (12% SDS-PAGE) under reducing and non-reducing conditions.

### Proteolytic analysis of the extract

The proteolytic activity of the *P. semirufa* caterpillar bristle extract was evaluated using fluorescence resonance energy transfer (FRET) peptide substrates, with readings performed in a spectrofluorometer. For this, the peptide Abz-FRSSRQ-EDDnp was dissolved in 10% dimethyl sulfoxide (DMSO - Merck, Darmstadt, Germany) and then diluted in Milli-Q water to allow the use of volumes where the concentration of the organic solvent did not exceed 5% of the final incubation volume (100 µL). For the assay, samples of the extract (1.0 µg) were incubated with peptide samples (5.0 µM) in buffered saline solution (PBS, pH 7.4) using 96-well plates and analyzed in a spectrofluorometer (FLUOstar Omega, BMG Labtech Inc, Durham, NC, USA) with excitation and emission wavelengths of 320 and 420 nm, respectively. The reaction temperature was maintained at 37 °C in a thermo-stabilized compartment under agitation. The increase in fluorescence was monitored continuously for 15 minutes, and the specific proteolytic activity of the extracts was expressed as µM of substrate cleaved per minute per microgram of extract (U/µg), calculated using the formula: Hydrolysis rate (µM/min)/[prot] (µg).

### Chondrocyte culture

Human chondrocytes, derived from normal human articular cartilage, were obtained from Lonza (Walkersville, MD, USA). For culturing and expanding the monolayer cell culture, a vial of chondrocytes, at a concentration of 10^6^ cells/mL, was thawed for approximately 1.5 minutes in a water bath and quickly transferred to a 75 cm^2^ cell culture flask containing 15 mL of chondrogenic growth medium supplemented with growth factors and 10% fetal bovine serum (FBS; Lonza, Walkersville, MD, USA). The flask was then transferred to an incubator at 37°C and 5% CO_2_ for cell growth. Once confluence was reached, the culture was trypsinized and transferred to other flasks for continued cell expansion up to the third passage. The cells obtained were frozen and stored in liquid nitrogen.

For subsequent experiments, the medium was gradually replaced with DMEM/F12 medium (Sigma-Aldrich, San Luis, MO, USA) supplemented with 14 mM sodium bicarbonate, 1% Penicillin-streptomycin solution, and 10% FBS, reaching full transition by the 5^th^ passage, when they were plated in 24- or 96-well plates at a density of 1.2×10^5^ and 1.2×10^4^ cells/ well, respectively. Different concentrations of *P. semirufa* bristle extract (15 and 60 µg/mL *per* well) were added, and the chondrocytes were cultured in DMEM/F12 medium containing 1% FBS for 15 and 30 minutes, and 1, 6, and 24 hours. As a negative control, the chondrocytes were cultured in the presence of the same volume of PBS, and as a positive control, IL-1β (R&D Systems, Minneapolis, MN, USA) (10 ng/mL) was used. The culture was maintained in an incubator at 37°C, 5% CO_2_. At the end of each period, the supernatant was removed, centrifuged at 2,200 rpm for 20 minutes at 4°C, aliquoted, and frozen at −80°C for later analysis. In parallel, the adhered cells were assessed for proliferation/viability by MTT assay, cell lysate for western-blot assay or RNA extraction for RT-qPCR assay.

### Cell viability analysis by MTT method

The proliferation/viability of the adhered cells was evaluated using the MTT assay [23], which is based on the absorption of MTT salt (3-(4,5-dimethylthiazol-2-yl)-2,5-diphenyltetrazolium bromide, Sigma, San Luis, MO, USA) by viable cells and spectrophotometric determination of the product formed. After the treatments, i.e., incubation of the cells in the presence or absence of *P. semirufa* bristle extract (15 and 60 µg/mL per well) for 1, 6 and 24 hours, the cells were incubated with 0.5 mg/mL of MTT in a volume of 100 µL for 3 hours at 37°C in a 5% CO_2_ incubator. During this period, the MTT is taken up by viable cells (metabolically active cells) and reduced, forming formazan crystals that accumulate in the cytoplasm. After dissolving the crystals with dimethyl sulfoxide (DMSO) (Merck, Darmstadt, Germany) in the same volume (100 µL) for 10 minutes, the absorbance of the solution was measured at a wavelength of 540 nm using a spectrophotometer (FLUOstar Omega, BMG Labtech Inc, Durham, NC, USA).

### Preparation of whole cell lysate

Human chondrocytes, cultured in 24-well plates at a cell density of 1.2 × 10^5^ cells/well, treated with PBS buffer, IL-1β, or *P. semirufa* bristle extract (60 µg/mL) for 24 hours, were trypsinized and pooled (8 wells *per* biological replicate). After centrifugation, the pellet was resuspended in 150 µL of RIPA buffer (Tris HCl 10 mM, EDTA 1 mM, NaCl 150 mM, SDS 0.1%, Triton X-100 1%) with protease inhibitors (Roche 11836153001, Basel, Switzerland). The lysates were incubated on ice for 30 minutes with vortexing every 10 minutes. The samples were then centrifuged to remove insoluble cellular debris at 12,000 × g for 5 minutes at 4°C. The supernatants (total cell lysate) were collected and stored at −80°C until further use. The protein concentration of the lysates was determined using the BCA assay (Protein Assay Kit, Pierce Biotechnology, Inc., Waltham, MA, USA) following the manufacturer’s instructions.

### Analysis of the expression of NF-κB, IκB-α, p-IκB-α, and GAPDH by western blot

The presence of NF-κB, IκB-α, p-IκB-α, and GAPDH was assessed by western blot in cell lysates of chondrocytes treated with PBS buffer, IL-1β, or *P. semirufa* bristle extract (60 µg/mL). Equal amounts of protein from each treatment (10 µg) were electrophoresed on a 10% SDS-PAGE gel under reducing conditions. The components present in the gel were electrotransferred to nitrocellulose membranes. After transfer, the membranes were blocked with PBS-BSA 5% and incubated with antibodies against NF-κB (sc-8008, Santa Cruz Biotechnology, Dallas, TX, USA – diluted 1:200), p-IκB-α (sc-8404, Santa Cruz Biotechnology, Dallas, TX, USA – diluted 1:200), IκB-α (sc-1643, Santa Cruz Biotechnology, Dallas, TX, USA – diluted 1:200), and GAPDH (ab9485, Abcam, Cambridge, UK – diluted 1:200) for 1 hour with agitation. The membranes were washed with PBS-Tween 0.05%, and then incubated with specific secondary antibodies (anti-Rabbit IgG conjugated with AP, S3731, Promega, Madison, WI, USA – diluted 1:7500, and anti-Mouse IgG conjugated with AP, S372B, Promega, Madison, WI, USA – diluted 1:7500) for 1 hour with agitation. The membranes were then rewashed, and the reaction was developed by adding NBT and BCIP (Promega, Madison, WI, USA) according to the manufacturer’s recommendations. Densitometric analysis was conducted using ImageJ software to quantify the band intensity of target proteins relative to GAPDH, allowing for normalization of protein expression levels across samples.

### RNA extraction and cDNA synthesis

Human chondrocytes were cultured in 24-well plates and treated with buffer, IL-1β or *P. semirufa* bristle extract (60 μg/mL) for 1, 6 and 24 hours. The cell medium was then replaced with TRIzol (Invitrogen, Thermo Fisher Scientific Inc., Waltham, MA, USA) for RNA extraction according to the manufacturer’s recommendations, totaling 3 x 10^6^ cells in triplicate. Total RNA was analyzed using a Nanodrop One spectrophotometer (Thermo Scientific, Thermo Fisher Scientific Inc., Waltham, MA, USA). RNA concentration was determined at 260 nm, and quality was assessed based on the 260/280 nm ratio (≥1.8 was accepted as pure). Following the manufacturer’s recommendations, total RNA was treated with DNase I (Invitrogen, Thermo Fisher Scientific Inc., Waltham, MA, USA) to eliminate contaminating genomic DNA. Reverse transcription to complementary DNA (cDNA) was performed using 1,200 ng of treated RNA as a template in a reaction volume of 20 µL containing 2.5 µM oligo (dT) 12–18, 0.5 mM dNTPs, 5 mM DTT, 2 units of RNaseOUT Ribonuclease, and 10 units of SuperScript III reverse transcriptase, following the manufacturer’s instructions (Invitrogen, Thermo Fisher Scientific Inc., Waltham, MA, USA). All cDNA samples were stored at −20 °C for further quantitative real-time PCR (RT-qPCR) analysis.

### Real-time quantitative PCR

For RT-qPCR, two μL of cDNA, diluted eight times, 250 nM of each primer (forward and reverse; Integrated DNA Technologies, Coralville, Iowa, USA; S1 Table), and SYBR green (used according to the manufacturer’s recommendations; Applied Biosystems, Thermo Fisher Scientific Inc., Waltham, MA, USA) were used, totaling a volume of 10 μL.

The RT-qPCR was performed on the QuantStudio 3 Real-Time PCR system (Thermo Fisher Scientific, Waltham, MA, USA) with thermal cycling parameters consisting of a 3-minute pre-incubation at 95 °C, followed by a two-step amplification program of 40 cycles, which included a denaturation step at 95 °C for 3 seconds and an annealing/ extension step at 60 °C for 30 seconds. A melting curve analysis was conducted in the end of each run to validate product amplification. Standard curves, showing the dilution of cDNA in relation to the cycle threshold (Ct) values and the amplification efficiency, was calculated using the QuantStudio Design & Analysis Software, version 1.5.1 (Applied Biosystems, Thermo Fisher Scientific Inc., Waltham, MA, USA). Only primers with amplification efficiency between 90 and 110 percent were accepted. Transcription levels were normalized to *GAPDH* and *RPL13A* (using the geometric mean between them), and the results were presented as relative abundance using the 2–ΔΔCT method.

### Cytokine levels in the supernatant of chondrocytes treated with extract from the bristles of *P. semirufa*

The concentration of cytokines in the supernatants of cultures treated with *P. semirufa* bristle extract, PBS buffer, or IL-1β, collected and frozen at −80°C at different time points, was evaluated by flow cytometry using the BD Biosciences kit (New Jersey, PA, USA): BD Cytometric Bead Array (CBA) Human Inflammatory Cytokines, following the manufacturer’s recommendations. The samples were analyzed for the presence of IL-6, IL-8, IL-10, IL-12, TNF, and IL-1β. The concentration of each factor was determined using the FCAP Array 3.0 software (BD Biosciences, New Jersey, PA, USA).

### Statistical analyses

Statistical analyses were performed using GraphPad Prism software (San Diego, CA, USA). To assess significant differences between the evaluated groups, ANOVA was used followed by the Tukey’s test, and t-test were used. For these analyses, the confidence interval was set at 95%, and statistical significance was considered when p < 0.05.

## Results

### Analysis of the cellular viability of human chondrocytes in the presence *P. semirufa* bristle extract

The protein concentration in the Pararama bristle extract prepared for use in chondrocyte cultures was 349.862 µg/mL, and the proteolytic activity was 11.76 ± 0.3819 U/min/µg. Electrophoretic separation of the bristle extract revealed a complex mixture of various proteins with molecular weights ranging from 17 to 200 kDa (S1 Fig).

**Fig 1:**
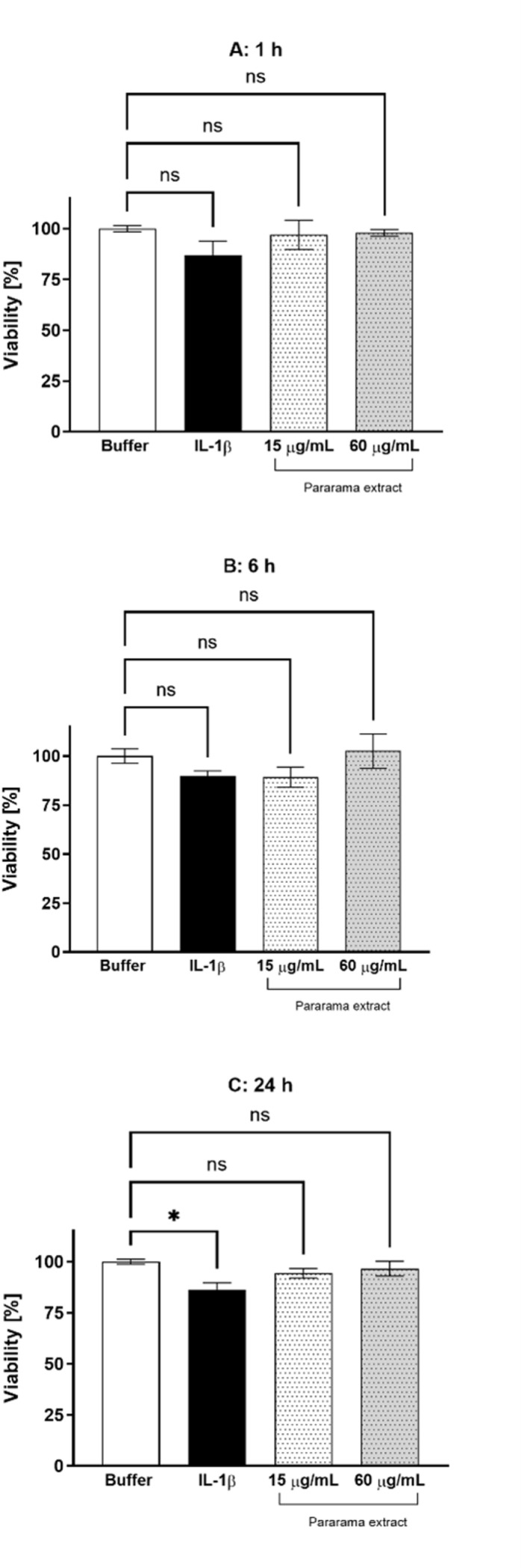
Viability of chondrocytes after treatment with *P. semirufa* bristle extract. Human chondrocytes were cultured in 96-well plates at a concentration of 7.5 × 10^4^ cells/mL and treated with bristle extract at concentrations of 15 and 60 µg/mL for periods of 1 (A), 6 (B), and 24 (C) hours. Cell viability was assessed using the MTT assay. Data are representative of two experiments conducted in triplicate, with results expressed as the mean of replicates ± standard error of percentage cell viability. (*) indicate *p* < 0.05 when treatments with the extract were compared to the positive control (IL-1β) and negative control (PBS), respectively, using One-way ANOVA followed by Tukey’s multiple comparisons test.

The viability of chondrocytes was assessed after treatment with different concentrations of bristle extract (15 and 60 µg/mL *per* well) for periods of 1, 6, and 24 hours. Fig 1 shows that the extract did not alter cell viability at either concentration or at any of the tested time points. The positive control, IL-1β, at the 24-hour time point, caused a slight but significant reduction in the viability of human chondrocytes compared to the control samples.

### Western blot analysis of NF-κB and IκB expression in human chondrocytes treated with *P. semirufa* bristle extract

**Fig 2:**
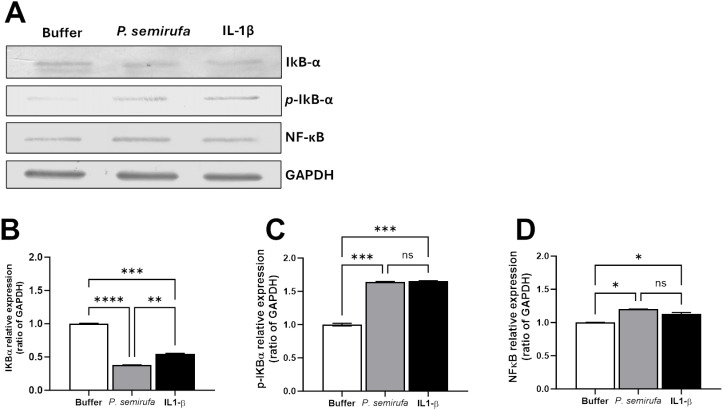
Western Blot and densitometric analysis of NF-κB, IκB-α, p-IκB-α, and GAPDH in chondrocytes. Chondrocytes were treated with PBS buffer, IL-1β (10 ng/mL), or Pararama bristle extract (60 µg/mL). (A) Total protein extracts were prepared using RIPA lysis buffer containing protease inhibitors. Protein lysates (10 µg) were separated by 10% SDS-PAGE and transferred to nitrocellulose membranes. After blocking with PBS-BSA 5%, membranes were incubated with primary antibodies against IκB-α p-IκB-α, NF-κB, and GAPDH (all diluted 1:200). Following washes, membranes were treated with secondary antibodies (diluted 1:7500) and developed using NBT and BCIP (Promega, USA). GAPDH served as a loading control. (B-D) Densitometric analysis was conducted using ImageJ software to quantify the band intensity of target proteins relative to GAPDH. Data were analyzed using one-way ANOVA and Tukey’s post hoc test, with significance levels indicated as (*) *p* ≤ 0.05, (**) *p* ≤ 0.01, (***) *p* ≤ 0.001, and (****) *p* ≤ 0.0001.

Fig 2 presents the Western blot and densitometric analyses of cellular lysates from human chondrocytes, assessing the presence of NF-κB, native IκB-α, phosphorylated IκB-α (p-IκB-α), and GAPDH (as a protein loading control). The cells were treated with *P. semirufa* bristle extract at a concentration of 60 µg/mL, along with a negative control (PBS buffer) and a positive control (IL-1β at 10 ng/mL) for 24 hours. The results indicate that the different treatments did not significantly alter the expression levels of NF-κB or GAPDH. However, the treatment with *P. semirufa* extract or IL-1β resulted in more intense bands of phosphorylated IκB proteins. Conversely, the presence of native IκB proteins was more pronounced in the negative control (buffer) treatment group. These findings suggest that *P. semirufa* bristle extract and IL-1β stimulate the phosphorylation of IκB, leading to its dissociation from NF-κB and the subsequent activation of the NF-κB pathway in human chondrocytes. The increased levels of phosphorylated IκB in the treated groups compared to the buffer control indicate that the extract contains components that can activate this signaling cascade.

### Gene expression modulation in human chondrocytes treated with *P. semirufa* bristle extract

**Fig 3:**
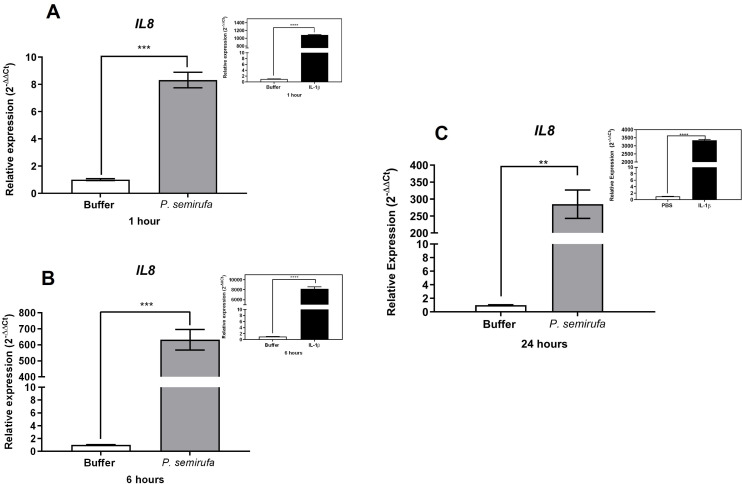
Induction of *IL8* gene expression in human chondrocytes treated with *P. semirufa* bristle extract is time- and dose-dependent. Human chondrocytes (1 × 10^5^ cells/well, four wells per biological replicate) were treated with PBS buffer, IL-1β, or bristle extract (60 μg/mL) for 1 (A), 6 (B) and 24 (C) hours. The cells were then collected, and total RNA was extracted using TRIzol. Relative mRNA quantification was performed using RT-qPCR. All experiments were conducted in biological triplicates and technical duplicates, with values presented as the mean ± SEM normalized to *GAPDH* and *RPL13A* as endogenous controls. Data were analyzed using an unpaired t-test, with significance levels indicated as (**) *p < 0.01*, and (***) *p < 0.001*.

**Fig 4:**
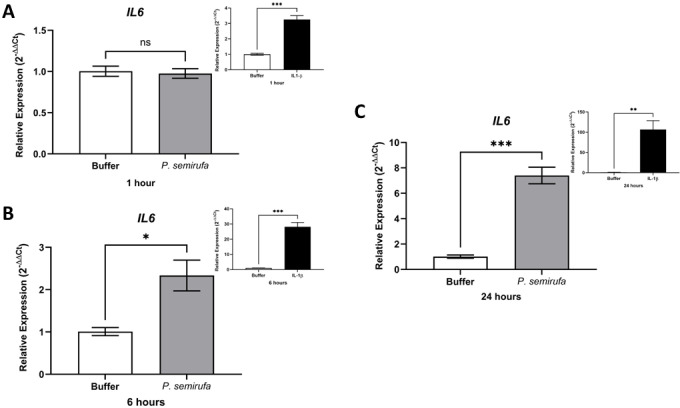
Induction of *IL6* gene expression in human chondrocytes treated with *P. semirufa* bristle extract is time- and dose-dependent. Human chondrocytes (1 × 10^5^ cells/well, four wells per biological replicate) were treated with PBS buffer, IL-1β, or bristle extract (60 μg/mL) for 1 (A), 6 (B) and 24 (C) hours. The cells were then collected, and total RNA was extracted using TRIzol. Relative mRNA quantification was performed using RT-qPCR. All experiments were conducted in biological triplicates and technical duplicates, with values presented as the mean ± SEM normalized to *GAPDH* and *RPL13A* as endogenous controls. Data were analyzed using an unpaired t-test, with significance levels indicated as (*) *p* *<* *0.05,* (**) *p < 0.01*, and (***) *p < 0.001*.

**Fig 5:**
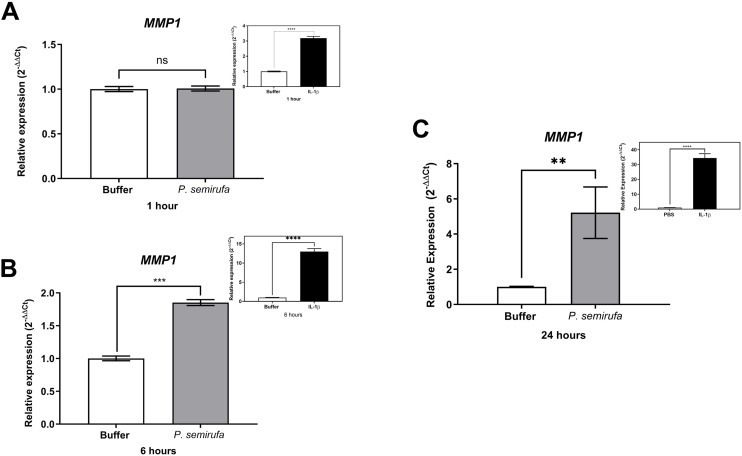
Increased gene expression of *MMP1* in chondrocytes treated with *P. semirufa* bristle extract. Human chondrocytes (1x10^5^ cells/well, four wells *per* biological replicate) were treated with PBS buffer, IL-1β, or bristle extract (60 μg/mL) for 1 (A), 6 (B) and 24 (C) hours. Following treatment, cells were collected, and total RNA was extracted using TRIzol. Relative mRNA quantification was performed via RT-qPCR. All experiments were conducted in biological triplicates and technical duplicates, with values presented as the mean ± SEM normalized to *GAPDH* and *RPL13A* as endogenous controls. Data were analyzed using an unpaired t-test, with significance levels indicated as (**) *p < 0.01* (***), *p < 0.001,* and (****) *p < 0.0001*.

**Fig 6:**
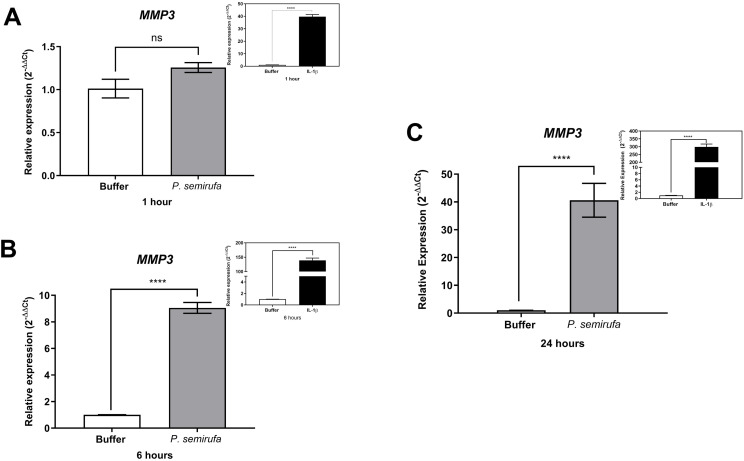
Increased gene expression of *MMP3* in chondrocytes treated with *P. semirufa* bristle extract. Human chondrocytes (1 × 10^5^ cells/well, four wells *per* biological replicate) were treated with PBS buffer, IL-1β, or bristle extract (60 μg/mL) for 1 (A), 6 (B) and 24 (C) hours. Following treatment, cells were collected, and total RNA was extracted using TRIzol. Relative mRNA quantification was performed via RT-qPCR. All experiments were conducted in biological triplicates and technical duplicates, with values presented as the mean ± SEM normalized to *GAPDH* and *RPL13A* as endogenous controls. Data were analyzed using an unpaired t-test, with significance levels indicated as (****) *p < 0.0001*.

**Fig 7:**
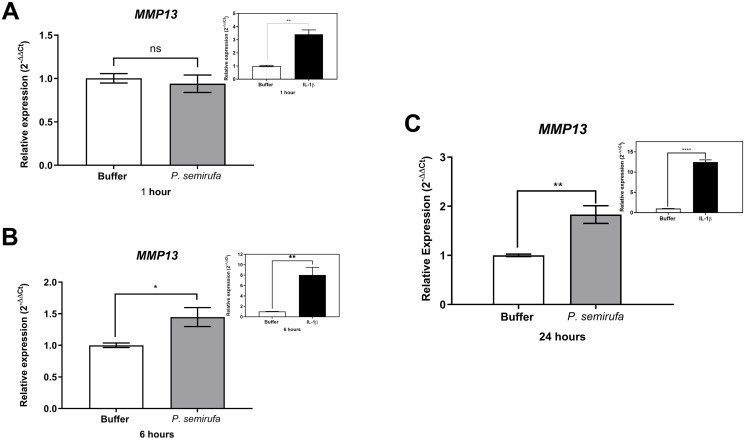
Increased gene expression of *MMP13* in chondrocytes treated with *P. semirufa* bristle extract. Human chondrocytes (1 × 10^5^ cells/well, four wells per biological replicate) were treated with PBS buffer, IL-1β, or bristle extract (60 μg/mL) for 1 (A), 6 (B) and 24 (C) hours. Following treatment, cells were collected, and total RNA was extracted using TRIzol. Relative mRNA quantification was performed via RT-qPCR. All experiments were conducted in biological triplicates and technical duplicates, with values presented as the mean ± SEM normalized to *GAPDH* and *RPL13A* as endogenous controls. Data were analyzed using an unpaired t-test, with significance levels indicated as (*) *p < 0.05* (**), *p < 0.01,* (***) *p < 0.001,* and (****) *p < 0.0001*.

**Fig 8:**
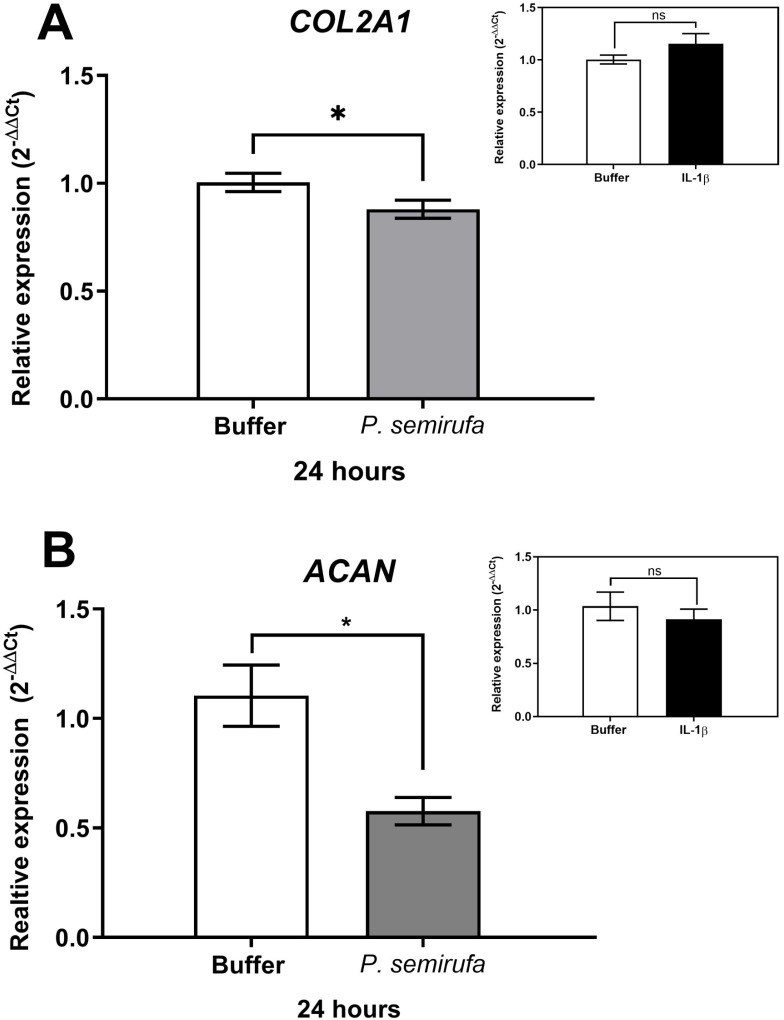
Reduction in gene expression of *ACAN* and *COL2A1* in chondrocytes treated with *P. semirufa* bristle extract. Human chondrocytes (1x10^5^ cells/well, four wells per biological replicate) were treated with PBS buffer, IL-1β, or bristle extract (60 μg/mL) for 24 h. After treatment, cells were collected, and total RNA was extracted using TRIzol. Relative mRNA quantification of *ACAN* (A) and *COL2A1* (B) was performed by RT-qPCR. All experiments were conducted in biological triplicates and technical duplicates, with values presented as the mean ± SEM normalized to *GAPDH* and *RPL13A* as endogenous controls. Data were analyzed using an unpaired t-test, with significance levels indicated as (*) *p < 0.05.*

**Fig 9:**
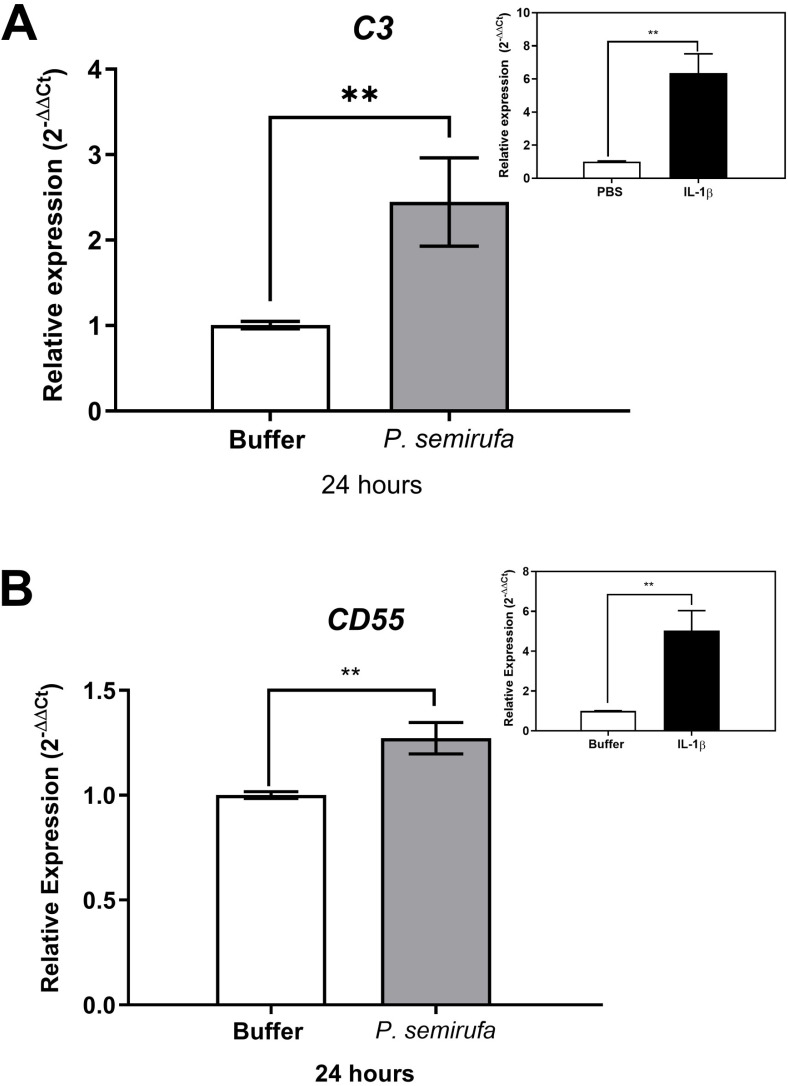
Induction of *C3* and *CD55* gene expression in human chondrocytes treated with *P. semirufa* bristle extract. Human chondrocytes (1 × 10^5^ cells/well, four wells per biological replicate) were treated with PBS buffer, IL-1β, or bristle extract (60 μg/mL) for 24 hours. After treatment, cells were collected, and total RNA was extracted using TRIzol. Relative mRNA quantification of *C3* (A) and *CD55* (B) was performed by RT-qPCR. All experiments were conducted in biological triplicates and technical duplicates, with values presented as the mean ± SEM normalized to *GAPDH* and *RPL13A* as endogenous controls. Data were analyzed using an unpaired t-test, with significance levels indicated as (**) *p < 0.01*.

We also evaluated the gene expression in human chondrocytes treated with *P. semirufa* bristle extract, IL-1β, or buffer. Fig 3 shows that after a 1-hour treatment, only *IL8* expression was significantly increased in chondrocytes treated with the Pararama extract compared to the PBS control. Similarly, IL-1β also induced a marked increase in *IL8* expression. As the treatment duration increased to 6 hours, a wider range of genes exhibited altered expression. Specifically, *IL6*, *IL8*, *MMP1*, *MMP3*, and *MMP13* were positively regulated in chondrocytes treated with the *P. semirufa* bristle extract compared to those receiving PBS (Figs 3–7). Additionally, IL-1β also induced a significant increase in *IL6*, *IL8*, *MMP1*, *MMP3*, and *MMP13* expression. Further analysis conducted after 24 hours of treatment revealed intricate changes in gene expression. Notably, there was a significant reduction in the expression of aggrecan (*ACAN*) and collagen type II (*COL2A1*), both of which are critical components of cartilage. This reduction suggests a potential shift towards cartilage degradation (see Fig 8). In contrast, the expression levels of interleukins (*IL6* and *IL8*), complement system molecules (*C3* and *CD55*), and extracellular matrix molecules (*MMP1*, *MMP3*, and *MMP13*) increased significantly compared to the negative control (Buffer). Additionally, IL-1β was found to induce a substantial increase in the expression of *IL6*, *IL8*, *MMP1*, *MMP3*, *MMP13*, *C3*, and *CD55* (Figs 3–9).

It is important to note that expressions of *ACAN*, *COL2A1*, *C3*, and *CD55* were also evaluated at 1 and 6 hours of treatments; however, no modulation was observed during these time points for any of these genes.

### Time- and dose-dependent induction of pro-inflammatory cytokines IL-6 and IL-8 by *P. semirufa* bristle extract in human chondrocytes

**Fig 10:**
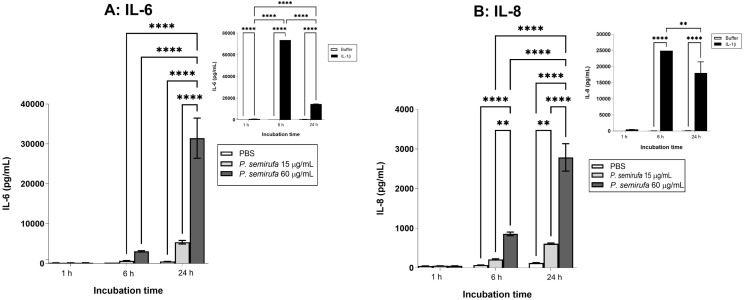
Cytokine production in the supernatant of chondrocytes treated with *P. semirufa* caterpillar bristle extract. Chondrocytes were cultured in a 96-well plate at a concentration of 7.5 x 10^4^ cells/mL and treated with two concentrations of *P. semirufa* bristle extract, 15 and 60 μg/mL per well, for periods of for 1, 6 and 24 hours. At the end of each period, the supernatants were removed, centrifuged at 2,200 rpm for 20 minutes at 4 °C, aliquoted, and assessed for cytokine concentration by flow cytometry. The data represent an experiment performed in triplicate, and the results were expressed as the mean of the triplicates ± SEM of the molecule concentrations under study. (**) *p <0.01* and (****) *p* < *0.0001* when treatments were compared to the negative or positive controls, i.e., PBS and IL-1β, respectively, using the Two-way Anova and Tukey’s post hoc test. “A” correspond to IL-6 dosage and “B” correspond to IL-8 dosage.

Analysis of the production of pro-inflammatory mediators in the supernatants of human chondrocytes treated with *P. semirufa* bristle extract, IL-1β, or buffer shows significant findings. Fig 10 illustrates that the extract notably induced the production of IL-6 and IL-8 among the analyzed cytokines compared to the negative control (buffer), in a dose- and time-dependent manner. Furthermore, the positive control also resulted in significantly elevated levels of these cytokines.

## Discussion

Pararamosis is an occupational and pro-inflammatory disease resulting from accidental contact with the bristles of *P. semirufa*. This condition can lead to deformities due to osteoarticular alterations, affecting cartilage and presenting a clinical picture similar to chronic synovial arthritis. Despite its impact, little is known about the molecular mechanisms underlying the venom’s action contained in the bristles, and currently, there is no effective treatment for this disease.

The present study aimed to investigate the molecular characteristics of how *P. semirufa* bristle extract affects human chondrocytes – cells found in joints – especially in relation to the inflammatory response triggered by envenomation. Our findings revealed that treatment with *P. semirufa* extract did not affect cell viability under any of the tested conditions. This aligns with previous research indicating that the extract from pararama caterpillar bristles does not cause chondrocyte cell death [24]. Therefore, we can conclude that the inflammation and catabolic responses observed are not a result of cytotoxicity, emphasizing the specific inflammatory nature of this response.

The *P. semirufa* bristle extract is composed of a complex mixture of toxins that likely activate cellular targets and initiate an inflammatory cascade. Our group has previously identified both toxic and non-toxic proteins in the bristles extract of *P. semirufa* through proteomic analysis. These proteins have the potential to induce various biological processes, including inflammatory pathways, extracellular matrix remodeling, cellular stress responses, innate immunity, and neutrophil degranulation, all of which are commonly associated with joint diseases [25]. Interestingly, some molecules in the extract are homologous to human proteins, including aldo-keto reductases, which have been shown to directly influence NF-κB activation, promoting inflammation and regulating pro-inflammatory cytokines such as IL-6 [26].

Our previous transcriptomic results have identified NF-κB as a differentially expressed gene in human chondrocytes treated with *P. semirufa* bristle extract [24]. Given the pivotal role of NF-κB in the progression of osteoarthritis, we analyzed the expression of the transcription factor NF-κB and its inhibitory protein, IκB, at the protein level. Our results indicated that treatment with the extract did not alter NF-κB levels, as assessed after 24 hours of incubation. However, we observed significantly higher levels of phosphorylated IκB proteins compared to the negative control (PBS), while unphosphorylated IκB proteins were more abundant in the buffer treatment group. This mechanistic detail elucidates how the bristle extract promotes the phosphorylation of IκB proteins, facilitating the translocation of NF-κB complexes from the cytoplasm to the nucleus and thereby activating target genes.

NF-κB has been implicated in chondrocyte catabolism, survival, and synovial inflammation associated with osteoarthritis [27]. The activation of NF-κB has been identified as a key pathway responsible for expressing genes involved in inflammatory responses in arthritis, including those encoding inflammatory cytokines, chemokines, and MMPs, through a positive feedback loop that enhances NF-κB signaling [28–30]. Consequently, NF-κB can exacerbate joint damage by promoting tissue inflammation, synthesizing catabolic factors, and inducing apoptosis in articular chondrocytes. Given NF-κB’s critical role in osteoarthritis as a therapeutic target, several drugs aimed at inhibiting this signaling pathway are currently under development [30].

We also evaluated gene expression in human chondrocytes treated with *P. semirufa* bristle extract, IL-1β, or buffer as control. After a 1-hour treatment, only *IL8* gene expression was significantly increased in chondrocytes treated with the *P. semirufa* bristle extract, indicating an early inflammatory response to the extract. As the treatment duration was extended to 6 hours, additional genes exhibited increased expression. Among these, *IL6*, *IL8*, *MMP1*, *MMP3* and *MMP13* were positively regulated in chondrocytes treated with the Pararama extract. This suggests that the extract stimulates inflammatory pathways and also influences matrix remodeling in the early stages of envenomation, and these stimuli last for hours. IL-1β also significantly increased the expression of *IL6*, *IL8*, *MMP1*, *MMP3*, and *MMP13*.

After 24 hours of treatment, there was a marked decrease in the expression of aggrecan (*ACAN*) and collagen type II (*COL2A1*), which are essential components of articular cartilage [31,32]. Conversely, the gene expression levels of inflammatory cytokines (*IL6*, *IL8*) and matrix metalloproteinases (*MMP1*, *MMP3*, *MMP13*) were substantially increased compared to the negative control. These findings suggest that the bristle extract promotes a catabolic and inflammatory phenotype in chondrocytes, potentially leading to cartilage degradation. Furthermore, the potent pro-inflammatory stimulus provided by IL-1β also induced significant upregulation of *IL6*, *IL8*, *MMP1*, *MMP3*, and *MMP13*. This indicates that both the bristle extract and IL-1β can independently modulate the expression of genes involved in inflammation and extracellular matrix remodeling in human chondrocytes. The sustained upregulation of catabolic and inflammatory markers, along with the downregulation of *ACAN*, suggests that the *P. semirufa* bristle extract may promote an osteoarthritic-like phenotype in chondrocytes. This is consistent with the clinical manifestations of pararamosis, which include joint space narrowing, bone alterations, and degeneration of articular cartilage.

Given the critical role of the complement system in mediating pro-inflammatory responses, it is crucial to explore how components derived from bristles may interfere with this system. Previous studies conducted by our group have shown that extracts from pararama bristles contain enzymes capable of cleaving key complement components, specifically C3, C4, and C5. This enzymatic cleavage results in the generation of bioactive peptides, including anaphylatoxins, which are known to exert significant pro-inflammatory effects [13,33]. In our current study, we observed an increase in the gene expression of both C3 and CD55 in chondrocytes treated with the bristle extract.

C3 is a central molecule in the complement system, acting as a convergence point for all activation pathways. While normal C3 activation is essential for physiological functions, its abnormal activation is often linked to various complement-mediated diseases. Additionally, recent hypotheses suggest that C3 may play roles in apoptosis and angiogenesis [34,35]. The upregulation of C3 expression observed in this study may serve as a mechanism to replenish the component that could have been cleaved by the action of proteases present in the pararama bristle extract.

Decay-accelerating factor (DAF or CD55) primary function is to inactivate C3 convertases by dissociating them into their constituent proteins as well as preventing their assembly, which prevents formation of the membrane attack complex. This inhibitor has recently attracted substantial attention due to its role in various diseases [36]. Interestingly, a study by Hoek [37] found that deletion of CD55 improved arthritis symptoms in mouse models. This finding aligns with our observations of increased *CD55* expression in chondrocytes treated with the extract. Conversely, the increased expression of *CD55* may represent a cellular strategy to regulate and control the inflammatory response, highlighting an adaptive mechanism to prevent tissue damage during heightened complement activation induced by the extract. These results highlight the complex interplay between complement regulation and inflammatory responses in chondrocytes.

Our analysis also revealed that cytokine levels in the supernatants of chondrocytes treated with the bristle extract exhibited a significant increase in interleukin-6 (IL-6) and interleukin-8 (IL-8) after 24 hours of treatment, particularly at the highest concentration tested (60 µg/mL). The increase in IL-8 production was already observed after just 6 hours of treatment. IL-6 and IL-8 are well-known pro-inflammatory and angiogenic molecules, serving as potent chemoattractants for neutrophils. Numerous studies have shown that sustained elevations in these cytokines can lead to structural damage in cartilage, corroborating the results obtained in our study [38–40].

In conclusion, our research highlights that the *P. semirufa* bristle extract induces a pro-inflammatory response in human chondrocytes, characterized by the activation of NF-κB and increased expression of inflammatory cytokines and matrix metalloproteinases. These responses suggest a potential pathway through which the venom components contribute to cartilage degradation and inflammation, resembling the pathological features of osteoarthritis. Furthermore, the interaction between bristle components and the complement system may exacerbate these inflammatory processes, indicating a complex interplay that warrants further investigation.

Given the absence of effective treatments for pararamosis, our findings emphasize the need for continued research into the molecular interactions initiated by *P. semirufa* bristle exposure. Understanding these mechanisms could pave the way for developing targeted therapeutic strategies aimed at mitigating inflammation and preventing cartilage damage in affected individuals.

## Supporting information

S1 Fig Electrophoretic profile of P. semirufa bristle extract. Samples containing 10 μg of the extract from the bristles of *P. semirufa* were subjected to SDS-PAGE in a 12% polyacrylamide gel under non-reducing (A) and reducing (C) conditions. Panel (B) represents the molecular weight standard. The gel was stained using the silver impregnation method for protein visualization.
(DOCX)

S1 Table Sequence of primers used in RT-qPCR. (DOCX)
