# Supplementary material for: Investigating the impact of Premolis semirufa caterpillar bristle toxins on human chondrocyte activation and inflammation
Source: PLoS Negl Trop Dis. 2025 Feb 10;19(2):e0012816. doi: 10.1371/journal.pntd.0012816 (PMC11809898; doi:10.1371/journal.pntd.0012816)
Supplement: S1 Fig — Electrophoretic profile of P. semirufa bristle extract. Samples containing 10 μg of the extract from the bristles of P. semirufa were subjected to SDS-PAGE in a 12% polyacrylamide gel under non-reducing (A) and reducing (C) conditions. Panel (B) represents the molecular weight standard. The gel was stained using the silver impregnation method for protein visualization. (DOCX) [file pntd.0012816.s001.docx]

**
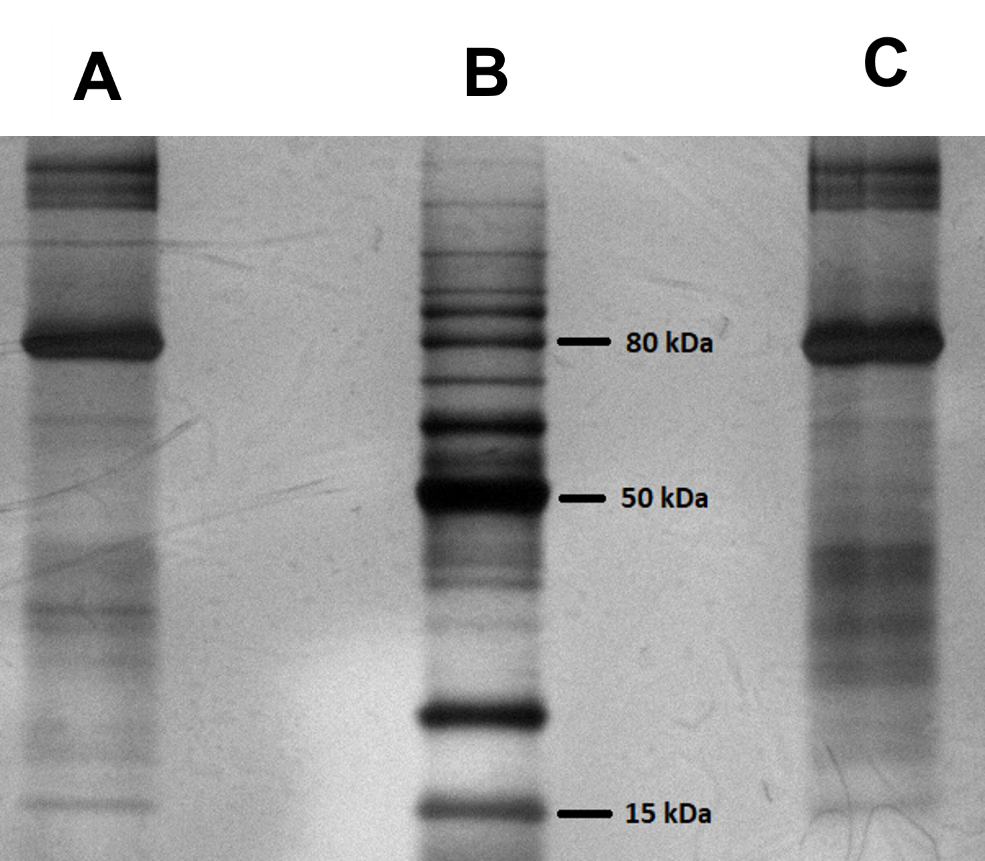
**

**S1 Fig. Electrophoretic profile of *P. semirufa* bristle extract.**Samples containing 10 μg of the extract from the bristles of *P. semirufa* were subjected to SDS-PAGE in a 12% polyacrylamide gel under non-reducing (A) and reducing (C) conditions. Panel (B) represents the molecular weight standard. The gel was stained using the silver impregnation method for protein visualization.
