# Supplementary material for: Investigating the impact of Premolis semirufa caterpillar bristle toxins on human chondrocyte activation and inflammation
Source: PLoS Negl Trop Dis. 2025 Feb 10;19(2):e0012816. doi: 10.1371/journal.pntd.0012816 (PMC11809898; doi:10.1371/journal.pntd.0012816)
Supplement: S1 Table — (DOCX) [file pntd.0012816.s002.docx]

**Table S1. Sequence of primers used in RT-qPCR**

| **Gene** | **NCBI acession number** | **Primer name** | **Primer sequence** |
| --- | --- | --- | --- |
| *GAPDH* | NM_002046.7 | Foward | CCCACTCCTCCACCTTTGAC |
|  |  | Reverse | CCACCACCCTGTTGCTGTAG |
| *RPL13A* | NM_012423.4 | Foward | GTATGCTGCCCCACAAAACC |
|  |  | Reverse | CTTCAGACGCACGACCTTGA |
| *C3* | NM_000064.4 | Foward | CTGCCCAGTTTCGAGGTCAT |
|  |  | Reverse | CAATCGGAATGCGCTTGAGG |
|  |  | Foward | CAATTGTGTCGCTGCCATCG |
| *CD55* | NM_001114752.3 | Reverse | ACCACCACACCAAATGCTCA |
|  |  | Foward | GGTGACAGGACGAGAACCAG |
|  |  | Reverse | ATACCCCGGTTCTTCTGCAC |
| *ACAN* | NM_001369268.1 | Foward | AAGACGGCTTCCACCAGTGT |
|  |  | Reverse | ATGCCATACGTCCTCACACC |
| *Col2A1* | NM_001844.5 | Foward | CCAGATGACCTTCCTACGCC |
|  |  | Reverse | TCAGGGCAGTGTACGTGAAC |
| *MMP1* | NM_002421.4 | Foward | CACGCCAGATTTGCCAAGAG |
|  |  | Reverse | TTGTCCCGATGATCTCCCCT |
| *MMP3* | NM_002422.5 | Foward | CACTCACAGACCTGACTCGG |
|  |  | Reverse | AAGCAGGATCACAGTTGGCT |
| *MMP13* | NM_002427.4 | Foward | GCCATTACCAGTCTCCGAGG |
|  |  | Reverse | TACGGTTGGGAAGTTCTGGC |
| *IL6* | NM_000600.5 | Foward | GTACATCCTCGACGGCATCTC |
|  |  | Reverse | TCACCAGGCAAGTCTCCTCAT |
| *IL8* | NM_000584.4 | Foward | CACCGGAAGGAACCATCTCAC |
|  |  | Reverse | GGCAAAACTGCACCTTCACAC |
